# Enrichment of Rare Mitochondrial DNA Variants Among Individuals With Kidney Disease Reveals Undiagnosed Mitochondrial Disease

**DOI:** 10.1016/j.ekir.2026.106578

**Published:** 2026-05-06

**Authors:** Daniel R. Schecter, Rory J. Tinker, Patrick O’Connell, Jiuhong Pang, Simon SzeKing Lee, Meltem Ece Kars, Aysegul Guvenek, Michael Preuss, Yuval Itan, Eva Morava, Tamas Kozicz, Michio Hirano, Ali Naini, Jaya Ganesh

**Affiliations:** 1 Department of Genetics and Genomic Sciences, Icahn School of Medicine at Mount Sinai, New York, New York, USA; 2 Department of Pathology and Cell Biology, Columbia University Irving Medical Center, New York, New York, USA; 3 Department of Artificial Intelligence and Human Health, Icahn School of Medicine at Mount Sinai, New York, New York, USA; 4 Department of Neurology, Columbia University Irving Medical Center, New York, New York, USA

**Keywords:** mitochondrial DNA, mitochondrial disease, population biobank, precision medicine, variant-level association

## Abstract

**Introduction:**

Pathogenic mitochondrial DNA (mtDNA) variants cause multisystem disease, yet their contribution to kidney disease remains incompletely characterized, partly because of exclusion of the mitochondrial genome from genetic studies.

**Methods:**

We evaluated mtDNA variation in 27,747 participants from the Mount Sinai Million Health Discoveries Program (MSM), an ancestrally diverse biobank with whole-exome sequencing and linked electronic health records (EHR). mtDNA variants were identified using MitoVerse and classified with MITOMAP. Kidney disease was defined using renal PheCodes for glomerular disease (GU_580) and renal failure (GU_582). Previous mitochondrial diagnoses were ascertained from EHR to identify undiagnosed individuals. Associations were adjusted for age, self-reported gender, and ancestry, with genotype–phenotype review.

**Results:**

Among 3935 individuals with kidney disease, 45 carried clinically associated mtDNA variants, 42 of whom had no previous clinical mitochondrial diagnosis. mtDNA variants were enriched among individuals with kidney disease and associated with increased odds of renal involvement (odds ratio [OR] = 1.72). Associations were strongest for chronic kidney disease (CKD; GU_582.2; OR = 1.55) and renal failure (GU_582; OR = 1.53). Among undiagnosed carriers, genotype–phenotype review identified concordant manifestations in 14%, including mitochondrial CKD with hyperuricemia. Variant-level analysis identified enrichment of m.1630A>G in *MT-TV* (OR = 5.56), with additional variants showing trends. Both renal- and nonrenal-associated pathogenic mtDNA variants were observed.

**Conclusion:**

Pathogenic mtDNA variants are overrepresented among individuals with kidney disease, often without a known mitochondrial diagnosis. These findings support a contributory role for mtDNA in renal disease and highlight the value of mtDNA analysis into kidney disease research and clinical evaluation, particularly for identifying unrecognized mitochondrial disease with renal involvement.

## Graphical abstract

**Figure:**
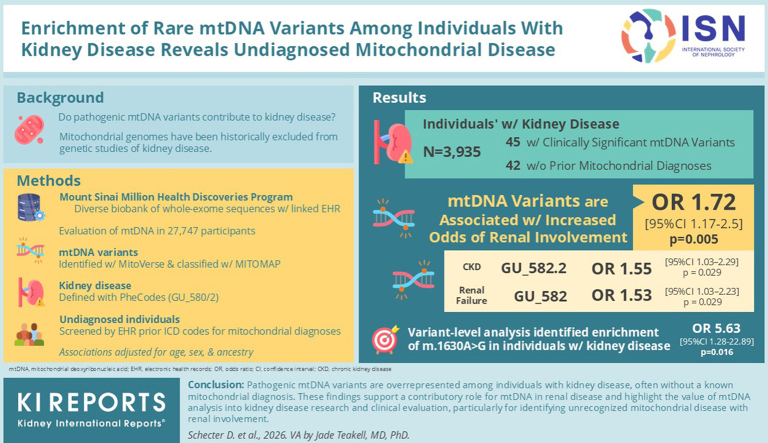

The mitochondria are organelles responsible for energy production, with additional roles in calcium homeostasis, metabolic regulation, apoptosis, cell signaling, and reactive oxygen species production. mtDNA encodes 37 genes— 22 transfer RNAs, 2 ribosomal RNAs, and 13 subunits of complex I, III, IV, and V of the electron transport chain.^1^ mtDNA replicates continuously and independently of cell division, with a mutation rate 5–10 times higher than nuclear DNA, which encodes approximately 1500 genes required for mitochondrial function, replication, and maintenance. Pathogenic variants in either genome can lead to mitochondrial dysfunction, referred to as primary mitochondrial disease.^2^^,^^3^ The prevalence of primary mitochondrial disease is about 1:5000; however, this is likely an underestimate because of diagnostic difficulty, wide variability in age of onset, and broad clinical spectrum.^2^ Diagnosis is further complicated by heteroplasmy, characterized by a mixture of wild-type and mutated mtDNA within a particular cell, with the degree and distribution of heteroplasmy across tissues determining phenotype and severity.^4^

Mitochondrial disease can affect almost any organ individually or present with multisystem involvement at any age.^4^ Although its neurological and cardiovascular effects have been well described, less is known about the renal manifestations and their prevalence.^5^ When adjusted for weight, the kidney is 1 of the highest energy-demand organs, rich in mitochondria given its roles in maintaining electrolyte balance, blood pressure, and filtration of waste products.^2^ Mitochondrial dysfunction is known to cause acute kidney injury, CKD, proximal and distal tubular dysfunction, nephrotic syndrome, and tubulointerstitial nephritis.^2^ Few publications comprehensively catalog the mtDNA variants linked to renal pathology, and major genomic studies of kidney disease—including the landmark exome-sequencing study by Groopman *et al.*^6^—excluded the mitochondrial genome entirely. Although recent population-based studies have linked common mtDNA variants to quantitative measures of kidney function, such as estimated glomerular filtration rate and serum creatinine and cystatin C, these analyses were not designed to evaluate established disease-causing mtDNA variants, assess the prevalence of undiagnosed primary mitochondrial disease, or leverage EHR-derived phenotyping required for primary mitochondrial disease diagnosis.^7^ These omissions contribute to persistent diagnostic gaps in both pediatric and adult kidney disease.

Given this under-recognition and the lack of dedicated mitochondrial genomic analysis in kidney disease, we evaluated mtDNA variation in patients with kidney disease, specifically glomerular disease and renal failure, within the MSM, of nearly 28,000 participants containing whole-exome sequencing samples and deidentified clinical information. MSM is among the most ancestrally diverse large-scale genomic biobanks, with more than 70% of participants classified as non-European genomic ancestry, including substantial representation of participants with African (27.9%), Amerindian (18.3%), East Asian (5.7%), and South Asian (4.3%) ancestry. This level of diversity exceeds that reported for many widely used biobanks, including the All of Us Research Program and the UK Biobank.

Our goal was to assess whether a subset of patients with kidney disease may, in fact, have an underlying primary mitochondrial disorder detectable through comprehensive mtDNA variant analysis and annotation. We further hypothesized that individuals carrying pathogenic mtDNA variants may have an increased risk of renal disease at the population biobank level and that the Mount Sinai Million cohort is uniquely positioned to address this question.

## Methods

### MSM & mtDNA Pipeline

MSM is a large-scale genomic biobank led by the Icahn School of Medicine at Mount Sinai in collaboration with the Regeneron Genetics Center. The program integrates genomic sequencing data with deidentified EHR from a large and ancestrally diverse patient population within the Mount Sinai Health System.

**Figure 1:**
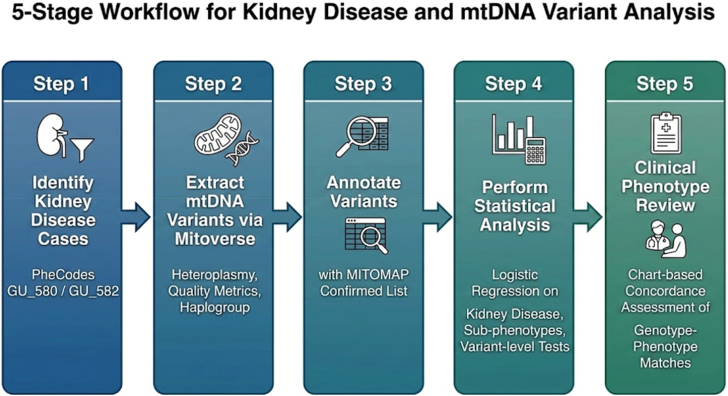
Overview of the analytic workflow for identifying and evaluating mtDNA variants in individuals with kidney disease. The 5-stage pipeline includes identification of kidney disease cases using renal PheCodes (GU_580 and GU_582), analysis of mtDNA derived from whole-exome sequencing using MitoVerse, variant annotation against the MITOMAP database to assess pathogenicity, statistical association testing at both the variant-carrier and variant-specific levels, and clinical phenotype review to assess genotype–phenotype relevance.

Whole-exome sequencing data from 27,747 MSM participants were analyzed using the Mitoverse mtDNA-Server 2 comprehensive mitochondrial bioinformatics pipeline (Figure 1).^8^ Mitoverse produced detailed variant annotations, including heteroplasmy levels, depth of coverage, base quality, genomic coordinates, and haplogroup assignment. A heteroplasmy detection threshold of 2% was applied, and no additional subsampling or variant allele frequency estimation tools were used.

All variants were cross-referenced with the MITOMAP list titled “ALL Disease Mutations with confirmed status.^9^” This list represents the most strictly curated clinically associated variant list within the MITOMAP disease variant database and, at the time of analysis, comprised 22 pathogenic (P), 86 likely pathogenic (LP), and 22 variants of uncertain significance. For this study, “clinically associated” mtDNA variants were defined as those included in this curated MITOMAP reference list, reflecting reported disease associations rather than American College of Medical Genetics and Genomics classification alone.

### PheCodes

To identify participants with kidney involvement potentially attributable to mitochondrial dysfunction, International Classification of Diseases (ICD)-9 and ICD-10 diagnosis codes from MSM participants were mapped to phecodeX.^10^ Filtering was then performed using renal phecodes, specifically GU_580 (glomerular disease) and its subphecodes (GU_580.1 acute nephritic syndrome; GU_580.2 chronic nephritic syndrome; GU_580.5 morphologic lesions of glomerular diseases; GU_580.51 focal and segmental glomerular lesions; GU_580.52 diffuse membranous glomerulonephritis; GU_580.53 diffuse mesangial proliferative glomerulonephritis; GU_580.54 diffuse endocapillary proliferative glomerulonephritis; GU_580.55 diffuse mesangiocapillary glomerulonephritis; GU_580.56 dense deposit disease; GU_580.57 diffuse crescentic glomerulonephritis; GU_580.58 C3 glomerulonephritis) as well as GU_582 (renal failure) and its subcategories (GU_582.1 acute kidney failure; GU_582.2 CKD; GU_582.21 end-stage renal disease [ESRD; CKD stage 5]; GU_582.3 renal dialysis).

Additional GU PheCodes associated with infections (e.g., pyelonephritis, urinary tract infection, cystitis), exposures (drug-induced or heavy metal toxicity), structural abnormalities (hydronephrosis, stricture, vesicoureteral reflux, renal cysts, small kidney), stones (renal colic, nephrolithiasis), laboratory findings (impaired renal function, hematuria), and nonspecific urinary symptoms (dysuria, incontinence, discharge) were excluded because of their lack of established association with primary mitochondrial disease.

### Population Sorting

For all participants within MSM, additional deidentified information was available, including family history, medical history, problem list based on ICD-9 and ICD-10 diagnoses, ICD-10 codes, encounter diagnosis type and year, and demographic variables (self-reported gender, ethnic group, country of origin, marital status, religion, patient-reported and self-reported race, genetic ancestry, and age).

Genetic ancestries of MSM participants were inferred using HapMap3 populations as a reference.^11^ Principal components were calculated from HapMap3 samples using common autosomal single nucleotide polymorphisms with a minor allele frequency > 5%, and MSM samples were projected onto these principal components. Kernel density estimates (KDEs) were then trained for each ancestral group using the first 4 principal components. Based on the KDE results, each MSM sample was assigned to 1 of 5 superpopulations according to its genetic similarity to the HapMap3 reference populations as follows: African (*n* = 7742), Amerindian (*n* =5087), East Asian (*n* = 1580), European (*n* = 10,580), or South Asian (*n* = 1197).

Pairwise relatedness was assessed using the Kinship-based inference for genome-wide association studies kinship relationship matrix with robust kinship coefficients among individuals in the renal cohort who carried known mitochondrial disease diagnoses or mitochondrial variants of interest.^12^ No related sample pairs were detected above the prespecified relatedness threshold; therefore, all samples were retained for analysis. Relationships were classified according to standard Kinship-based inference for genome-wide association studies thresholds, where kinship coefficients correspond to duplicate/monozygotic twin, first-degree, and second-degree relationships, respectively. Individuals related at the second degree or closer (kinship ≥ 0.0884) within this cohort would have been excluded.

### Statistics and Regression

Logistic regression was used to evaluate whether mtDNA variant carrier status was associated with kidney disease within the MSM cohort. For the primary analysis, we modeled the presence of any kidney disease PheCode (GU_580 or GU_582) as the outcome and carrier status of any variant included in the MITOMAP list titled “ALL Disease Mutations with confirmed status” as the main predictor. Participants without either GU_580 or GU_582 PheCodes served as controls. All models were adjusted for age, self-reported gender, and genetically derived ancestry, and standard errors were calculated using maximum likelihood estimation.

To further examine phenotype-specific patterns, targeted logistic regression analyses were conducted across each GU_580 and GU_582 sub-phenotype defined in the PheCodes section. Each subphenotype was modeled independently using the same covariate structure, and associations were interpreted in the context of case counts and sample size limitations.

Variant-level association testing was performed to assess whether specific mtDNA variants were enriched among individuals with kidney disease. Only the 42 mtDNA variants identified within the kidney disease cohort were included in this targeted variant-level analysis. Adjusted logistic regression was applied separately to each genotype present in the broader dataset (*n* ≈ 27,747), provided that sufficient carrier counts permitted model convergence. Variants with only 1 or 2 observed carriers were excluded because of instability of effect estimates. Odds ratios, 95% confidence intervals, and *P*-values were reported for all analyzable variants.

All statistical analyses were conducted using R in RStudio (version 2025.09.2+418, Posit Software PBC, Boston, MA). Significance was assessed using 2-sided tests without multiple-testing correction, given the targeted nature of the analyses and high correlation among investigated phenotypes.

## Results

### Analysis Reveals Kidney Disease Participants With Clinically Associated mtDNA Variants

**Figure 2:**
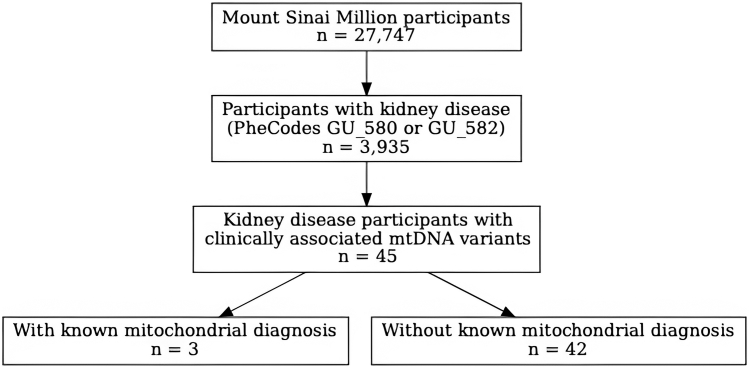
Cohort overview and identification of mtDNA-positive kidney disease participants. Sequential filtering of Mount Sinai Million participants identified 3,935 individuals with kidney disease (renal PheCodes GU_580/GU_582), including 45 individuals with a clinically associated mitochondrial DNA variant∗ or a known mitochondrial diagnosis. Of these, 3 had a documented mitochondrial diagnosis^†^, and 42 carried a clinically associated mtDNA variant without a previous diagnosis. Percentages are relative to the indicated parent cohort. ∗Clinically associated variants were defined as variants listed in the MITOMAP “All Disease Mutations with confirmed status” reference. ^†^Known mitochondrial diagnosis was determined based on problem list entries, PheCodes, or ICD-10 codes.

Filtering all of MSM (n ≈ 27,747) by renal PheCode, GU_580 and GU_582, identified 3935 individuals with kidney disease, of which 45 participants carried a clinically associated mtDNA variants (Figure 2). Of these, 3 individuals were identified through a clinical diagnosis of mitochondrial disease (GE_968), based on problem list entries, PheCodes, and ICD-10 codes. The remaining 42 individuals carried a variant listed in the MITOMAP “ALL Disease Mutations with confirmed status” list but did not have a known clinical diagnosis of mitochondrial disease based on PheCodes, problem list, and ICD-10 codes. To account for potential incomplete capture of tubular phenotypes, we searched the problem list for Fanconi syndrome and identified 1 individual within the total MSM cohort who also met renal PheCode inclusion criteria.

**Figure 3:**
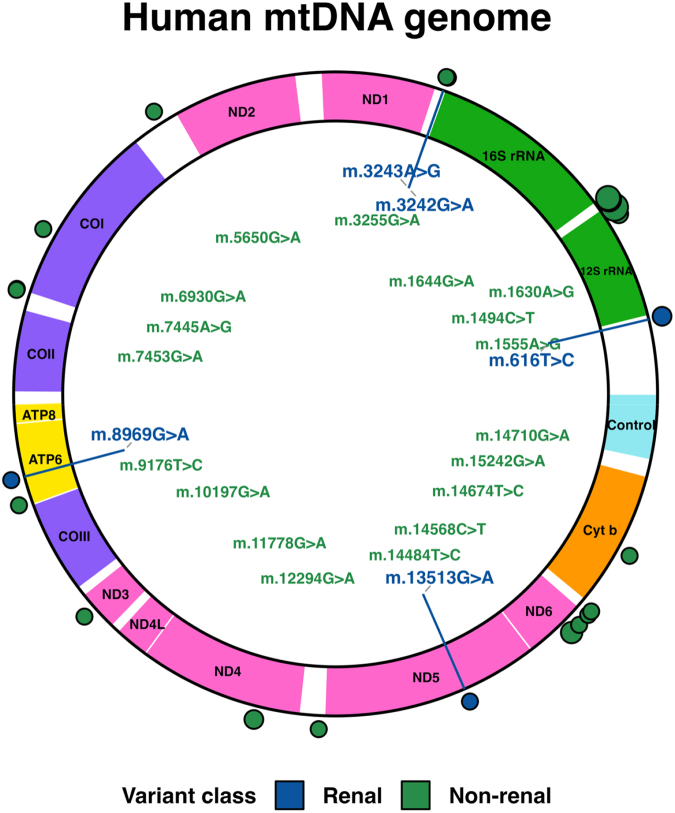
Distribution of variants identified in individuals with kidney disease across the mitochondrial genome. Variants carried by individuals within the kidney disease cohort are mapped to their genomic positions along the circular mitochondrial genome. Variants with previously reported renal manifestations are shown in blue, whereas variants without known renal associations are shown in green. All variants are drawn from the MITOMAP Disease Mutation List with Confirmed Status, illustrating the genomic distribution of clinically associated mtDNA variants across multiple mitochondrial loci within this cohort.

The 3 individuals with documented mitochondrial disease in our renal disease cohort, identified through the problem list and PheCodes, included 1 individual with mitochondrial encephalomyopathy, lactic acidosis, and stroke-like episodes, 1 with mitochondrial neurogastrointestinal encephalopathy syndrome, and 1 with mitochondrial myopathy. Among the 42 individuals without a known clinical mitochondrial diagnosis, 20 carried a pathogenic variant, 18 carried a likely pathogenic variant, and 6 carried a variant of uncertain significance, all included within the MITOMAP curated disease variant list (see Supplementary Table S1). Heteroplasmy levels ranged from low-level heteroplasmy (2.1%) to homoplasmy, with a mean heteroplasmy of approximately 74%; 9 variants were homoplasmic, and 15 demonstrated heteroplasmy levels ≥ 0.9. As seen in Figure 3, the mitochondrial variants were distributed across a broad range of loci, including multiple transfer RNA genes (e.g., *MT-TF, MT-TV, MT-TL1, MT-TA, MT-TS1, MT-TL2, MT-TE*), the 12S ribosomal RNA gene MT-RNR1, and several protein-coding genes spanning complexes I, III, IV, and V (e.g., *MT-ND3, MT-ND4, MT-ND5, MT-ND6, MT-CO1, MT-ATP6, MT-CYB*), reflecting involvement of multiple functional domains of the mitochondrial genome.

### Several mtDNA Variants Identified in This Cohort Have Reported Renal Manifestations

Clinically, many of the identified variants are associated with classical mitochondrial phenotypes such as myopathy, epilepsy, and Leigh syndrome, however, kidney manifestations have been previously reported in carriers of five variants identified in this cohort, including the following: m.616T>C in *MT-TF*, which causes mitochondrial tubulointerstitial kidney disease and Gitelman-like syndrome^13^, ^14^, ^15^; m.3242G>A in *MT-TL1*, associated with renal tubular dysfunction^16^; m.3243A>G in *MT-TL1*, linked to focal segmental glomerulosclerosis (FSGS)^17^^,^^18^; m.13513G>A in *MT-ND5*, reported in tubulointerstitial kidney disease^19^; and m.8969G>A in *MT-ATP6*, described in individuals with IgA nephropathy.^20^^,^^21^

### Positive mtDNA Variant Status is Enriched Among Individuals With Kidney Disease

Given the identification of multiple mtDNA variants in the Mount Sinai Million kidney disease cohort, we first performed a logistic regression to determine whether mtDNA-variant status was enriched among individuals with kidney disease compared with the remainder of the cohort. Of the 3935 individuals with kidney disease, 45 (1.1%) harbored at least 1 mtDNA variant included in the MITOMAP reference list, compared with 152 (0.6%) of the 23,812 individuals without kidney disease. Individuals positive for an mtDNA variant of interest had significantly higher odds of kidney disease (OR = 1.72, 95% CI: 1.17–2.50, *P* = 0.005) independent of age, genetically derived ancestry, and self reported gender.

### Specific Kidney Disease Sub-Phenotypes Demonstrate Targeted Associations With mtDNA Variant Status

To further characterize this signal, we conducted targeted logistic regression analyses across clinically relevant kidney disease sub-phenotypes, using the parent and sub-PheCodes of interest. mtDNA-variant status was significantly associated with the CKD sub-PheCode (GU_582.2; 3,374 cases; OR = 1.55, 95% CI: 1.03–2.29, *P* = 0.029) as well as the broader renal failure parent PheCode (GU_582; 3,861 cases; OR = 1.53, 95% CI: 1.03–2.23, *P* = 0.029), both supported by large case counts (see Supplementary Table S2). A nominal association was also observed with diffuse membranous glomerulonephritis (GU_580.52; 12 cases; OR = 13.27, CI: 0.72–68.88, *P* = 0.014), although this estimate was based on only 12 cases and should be interpreted with caution.

### Variant-Level Regression Identifies m.1630A>G (*MT-TV)* as Significantly Enriched in Kidney Disease

We next evaluated whether specific mtDNA variants identified among the 45 individuals with kidney disease and mtDNA variants of interest were enriched in the broader cohort. This variant-level analysis was restricted to the 23 unique mtDNA variants observed within this subgroup. Using the full MSM mtDNA variant dataset (n = 27,747 participants), we performed logistic regression analyses adjusted for age, genetically derived ancestry, and self reported gender for each targeted genotype. The m.1630A>G in *MT-TV* variant was observed in 4 individuals with kidney disease and 6 individuals without kidney disease, demonstrating a significant association with renal involvement (OR = 5.63, 95% CI: 1.28–22.89, *P* = 0.016).

**Figure 4:**
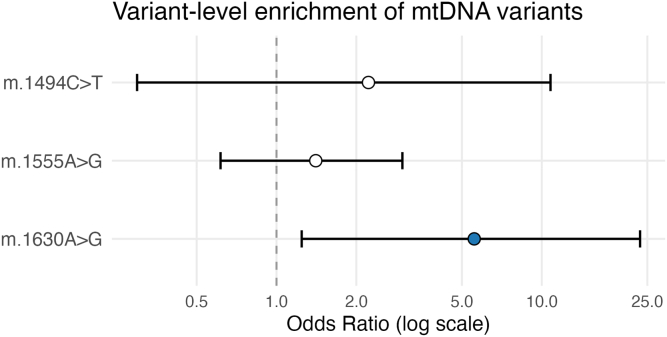
Variant-level enrichment of mitochondrial DNA variants in kidney disease. Forest plot of variant-level associations between selected mitochondrial DNA variants and kidney disease. Odds ratios and 95% confidence intervals were obtained from logistic regression models adjusted for age, self-reported gender, and genetic ancestry in the full MSM cohort.

Two additional variants showed modest but nonsignificant trends toward enrichment as follows: m.1555A>G (10 individuals with kidney disease, 37 without kidney disease; OR = 1.45, *P* = 0.39) and m.1494C>T (2 individuals with kidney disease, 8 without kidney disease; OR = 2.22, *P* = 0.36) (Figure 4). Of the remaining targeted variants, 5 were observed in only 1 individual and 5 were observed in 2 individuals, resulting in unstable model estimates and insufficient power for meaningful statistical interpretation. This includes m.616T>C, which was identified in 2 individuals with kidney disease and no individuals without kidney disease. Additionally, m.13513G>A, associated with tubulointerstitial kidney disease, identified in 2 cases (1 individuals with kidney disease, 1 without kidney disease; OR = 32.88, *P* = 0.029).^19^ Variants identified only in the renal cohort includes m.1644G>A, m.12294G>A, m.6930G>A, m.9176T>C, m.14710G>A, and m.15242G>A; however, these variants have no known renal phenotype and should be interpreted cautiously, as they may represent coincidental occurrences rather than disease-related associations.

### Clinical Review of Individual Cases Identifies Multiple Previously Unrecognized Mitochondrial Phenotypes

To better understand the clinical relevance of the 42 participants with kidney disease who carried a suspicious mitochondrial variant but had no previous diagnosis of mitochondrial disease, we evaluated genotype–phenotype concordance by assessing whether each individual’s documented medical history aligned with the known disease associations of their specific mtDNA variants. This assessment considered systemic clinical features to support the plausibility of the identified variants and was not limited to kidney-specific manifestations. Six of 42 individuals (14%) demonstrated strong genotype–phenotype concordance, meaning that their clinical features showed substantial overlap with the expected manifestations of the variant and that the observed heteroplasmy level was sufficient to support pathogenic expression (Table 1).

**Table 1:** Genotype–phenotype concordance among kidney disease cohort with mitochondrial DNA variants

Subject	mtDNA variant (gene)	MITOMAP classification	Heteroplasmy (%)	Overlapping clinical features observed	Associated disease
1	m.616T>C (MT-TF)	Likely pathogenic	5.10%	Chronic kidney disease; renal failure; elevated uric acid on laboratory assessment	Isolated chronic kidney disease; hyperuricemia
2	m.1494C>T (MT-RNR1)	Likely pathogenic	100%	Sensorineural hearing loss	Deafness
3	m.1555A>G (MT-RNR1)	Pathogenic	100%	Bilateral hearing loss	Antibiotic-induced and nonsyndromic hearing loss
4	m.1555A>G (MT-RNR1)	Pathogenic	99.20%	Bilateral hearing loss	Antibiotic-induced and nonsyndromic hearing loss
5	m.8969G>A (MT-ATP6)	Pathogenic / likely pathogenic (MLASA-associated)	22.9%	Sideroblastic anemia; musculoskeletal pain; acidosis; acid–base imbalance; chronic kidney disease	Mitochondrial myopathy, lactic acidosis, and sideroblastic anemia; reported IgA nephropathy
6	m.11778G>A (MT-ND4)	Pathogenic	100%	Blindness	Leber hereditary optic neuropathy

Shown are individuals within the kidney disease cohort whose documented systemic clinical features overlap with known disease associations of their respective mitochondrial DNA variants. This table highlights overall genotype–phenotype concordance within the cohort and is not limited to kidney-specific manifestations. Variant classification is based on MITOMAP, and heteroplasmy is reported as the percentage measured in blood.

This group included *MT-RNR1* deafness alleles, specifically m.1494C>T (LP) and m.1555A>G (P), as classified by MITOMAP, which serves as a member of the ClinGen mitochondrial DNA Variant Curation Expert Panel. For m.1494C>T, 1 of the 2 individuals had documented sensorineural hearing loss, and the variant was at homoplasmy.^22^ For m.1555A>G, 2 of 10 individuals exhibited sensorineural or bilateral hearing impairment with homoplasmy or near-homoplasmy (99%), consistent with *MT-RNR1*-associated nonsyndromic or aminoglycoside-sensitive hearing loss.^23^ Although hearing loss was not documented in the remaining carriers, 1 individual with m.1494C>T and 8 individuals with m.1555A>G, these individuals remain at increased risk, particularly with aminoglycoside exposure.

In addition, 1 participant with m.8969G>A (LP) in MT-ATP6 at 22.9% heteroplasmy exhibited a constellation of findings including sideroblastic anemia, musculoskeletal pain, acidosis, and CKD. The presence of sideroblastic anemia, musculoskeletal pain, and acidosis is consistent with the known phenotype of mitochondrial myopathy, lactic acidosis, and sideroblastic anemia.^20^ Previous reports have also described renal involvement, including IgA nephropathy, in association with this variant, suggesting a broader clinical spectrum, although nephropathy was not specifically documented in our case, but rather as CKD.^21^^,^^24^

Regarding the renal-specific variant m.616T>C (LP) in *MT-TF*, which has been associated with mitochondrial tubulointerstitial kidney disease and Gitelman-like syndrome, 2 individuals in the biobank carried this variant at low heteroplasmy levels (5.1% and 2.1%). Both had CKD and renal failure on their problem lists. There was no documentation of tubulointerstitial nephritis or Gitelman-like features in either individual; however, this variant has previously been reported in association with isolated CKD and hyperuricemia without neurological involvement at nonhomoplasmic levels.^14^ Notably, the individual with higher heteroplasmy (5.1%) had elevated uric acid, whereas uric acid testing was unavailable for the individual with lower heteroplasmy (2.1%).

Lastly, 1 participant with m.11778G>A (P) in *MT-ND4*, present at homoplasmy in blood, demonstrated blindness and low vision consistent with the Leber hereditary optic neuropathy (LHON) phenotype associated with this variant, which is the most common LHON variants.^25^^,^^26^ No alternative ophthalmic etiology was documented.

## Discussion

This study demonstrates the enrichment of disease-associated mtDNA variants among individuals with kidney disease and identifies a subset of patients carrying mtDNA variants with known renal manifestations, supporting an underlying mitochondrial contribution. Using comprehensive mtDNA variant analysis in a large, ancestrally diverse biobank, we identified clinically associated mtDNA variants both with and without known renal involvement in individuals with kidney disease, the majority of whom lacked a previous mitochondrial diagnosis. These variants spanned multiple regions of the mitochondrial genome, including coding and noncoding genes as well as transfer RNA and ribosomal RNA loci. At the population biobank level, mtDNA disease-associated variant status was associated with increased odds of kidney disease (OR = 1.72), particularly CKD (1.55) and renal failure (1.53), and variant-level analysis further identified enrichment of specific mtDNA variants (ex: m.1630A>G) among affected individuals. Collectively, these findings support our hypothesis that pathogenic mtDNA variants contribute to kidney disease in a subset of patients and demonstrate the value of incorporating mtDNA analysis into population-scale renal genomic studies and the investigation of kidney disease etiology.

Previous population-based studies have reported associations between mitochondrial genetic variation and kidney disease using aggregate or burden-based approaches.^27^ For example, an atlas of mitochondrial DNA genotype–phenotype associations in the UK identified numerous common mtDNA variants linked to a wide range of traits, including measures of renal function, using a phenotype-wide association framework.^7^ However, these studies primarily assessed global mtDNA variation and haplotypic effects across complex traits in the general population, rather than focusing on known pathogenic mtDNA variants with potential clinical implications. In contrast, our analysis specifically interrogates pathogenic and disease-causing mtDNA variants identified in individuals with clinically defined kidney disease, enabling direct genotype–phenotype correlations at the level of individual variants. This targeted approach moves beyond global burden estimation to assess the clinical relevance of established pathogenic mtDNA variants in kidney disease, thereby providing novel insights not captured by haplotype- or burden-based methods.

By integrating EHR-derived phenotypic data with variant-level association testing within a renal disease cohort, we identified 42 individuals with clinically associated mitochondrial variants and no formal diagnosis of a primary mitochondrial disorder. This included 3 individuals with deafness or bilateral sensorineural hearing loss, as well as an additional 9 individuals at high risk for hearing loss (either aminoglycoside- or nonaminoglycoside-related) based on their mtDNA variants. One individual with blindness carried a known pathogenic LHON variant at homoplasmy. Within the larger biobank, a renal-associated variant, m.616T>C, previously linked to isolated CKD and observed in both heteroplasmic and homoplasmic states, was identified in 2 individuals, both of whom had CKD and renal failure.^14^

This observation highlights the potential under-recognition of mitochondrial disease in routine clinical practice. Further work is needed to define the burden and clinical spectrum of undiagnosed mitochondrial disease across large biobank populations, including in individuals with nonrenal phenotypes.

Several limitations should be considered. These include the use of blood specimens rather than urine, as urine heteroplasmy levels correlate more closely with renal function than plasma and are often higher, whereas plasma heteroplasmy levels decrease most prominently with age.^28^^,^^29^ Additionally, this study was conducted using deidentified data without access to full patient charts, limiting the ability to definitively confirm concordance between mtDNA genotype and clinical phenotype. Although enrichment of disease-associated mtDNA variants was observed at the cohort level, the presence of a variant in an individual with kidney disease does not establish causality, and some co-occurrences may be coincidental. Caution is therefore warranted when interpreting individual variant–phenotype associations, particularly in the absence of consistent enrichment or functional validation.

An additional limitation relates to phenotyping of renal tubular dysfunction. Although mitochondrial disease commonly presents with tubulopathy, including renal Fanconi syndrome, there is no dedicated phecodeX classification for these phenotypes. Thus, our use of GU_580 and GU_582 may not capture isolated tubular dysfunction. To address this, we searched the problem list for Fanconi syndrome within the broader MSM cohort and identified 1 individual, who also met renal PheCode inclusion criteria and was included in the primary analysis.

Several variant-level mitochondrial renal associations cited in this study are based on limited case reports, often involving only 1 or 2 individuals, reflecting the rarity of these conditions and variants. Renal involvement in mitochondrial disease may reflect 1 component of a broader multisystem disorder rather than a primary manifestation, which is important to consider when interpreting these findings. Of note, within our cohort we did not identify any patients with single large scale mtDNA deletion syndromes, with prevalence of 1.2 in 100,000, such as Kearns-Sayre syndrome, where renal involvement is well characterized.^30^ Although our pipeline is capable of detecting mtDNA small insertions/deletions, historically, large-scale single mtDNA deletions are most reliably identified using Southern blotting or long-range polymerase chain reaction, which may limit detection in exome-based datasets.^8^

Future studies should address these limitations by performing more targeted mitochondrial assessments, both molecularly and clinically. These may include measurement of urine heteroplasmy levels, renal-specific laboratory testing or biopsy guided by variant-associated findings, and standardized clinical severity assessments such as the Nijmegen Pediatric Mitochondrial Disease Scale or the Newcastle Mitochondrial Disease Adult Scale to evaluate disease severity and multisystem involvement.^31^^,^^32^ Further functional studies are also needed to determine whether the presence of a disease-causing mtDNA variant alone is sufficient to impact renal function in the absence of a full syndromic phenotype, or whether the observed enrichment reflects previously unrecognized renal manifestations of known nonrenal pathogenic variants.

In conclusion, this study underscores both the utility and complexity of mtDNA analysis among individuals with kidney disease. It demonstrates the utilization and value of implementing mtDNA analysis within large biobanks, including 1 that did not initially incorporate mtDNA assessment but was able to integrate it through this project. Additionally, the identification of those with possible undiagnosed primary mitochondrial disease, both with and without known renal phenotype, highlights the importance of considering mtDNA analysis in cases of kidney disease of unknown etiology. As our understanding of the role and prevalence of mtDNA variants in kidney disease continues to evolve, these findings emphasize the clinical relevance of mitochondrial genomics in renal medicine. To our knowledge, this study is the first to demonstrate enrichment of disease-associated mtDNA variants in a kidney disease cohort compared with a nonkidney disease cohort, supporting a contributory role for pathogenic or disease-associated mtDNA variants in renal disease.

## Disclsoure

All the authors declared no competing interests.

## Supplementary Material

Supplementary File (PDF) **Table S1.** Mitochondrial DNA variants identified among kidney disease participants in the Mount Sinai Million cohort. **Table S2.** Kidney Disease Subtypes and mtDNA Carrier Frequency. STROBE Checklist.

## Footnotes

Supplementary File (PDF)
**Table S1.** Mitochondrial DNA variants identified among kidney disease participants in the Mount Sinai Million cohort. **Table S2.** Kidney Disease Subtypes and mtDNA Carrier Frequency. STROBE Checklist.
