# Supplementary material for: Enrichment of Rare Mitochondrial DNA Variants Among Individuals With Kidney Disease Reveals Undiagnosed Mitochondrial Disease
Source: Kidney Int Rep. 2026 May 6;11(7):106578. doi: 10.1016/j.ekir.2026.106578 (PMC13265648; doi:10.1016/j.ekir.2026.106578)
Supplement: Supplementary File (PDF) — Table S1. Mitochondrial DNA variants identified among kidney disease participants in the Mount Sinai Million cohort. Table S2. Kidney Disease Subtypes and mtDNA Carrier Frequency. STROBE Checklist. [file mmc1.pdf]

Supplement:

mtDNA variant (gene)	n	Heteroplasmy (%)	Reported disease associations	MITOMAP classification
m.616T>C (MT-TF)	2	5.1%, 2.1%	Maternally inherited epilepsy; mitochondrial tubulointerstitial kidney disease*; Gitelman-like syndrome*	Likely pathogenic
m.1494C>T (MT-RNR1)	2	100%, 100%	Deafness	Likely pathogenic
m.1555A>G (MT-RNR1)	13	100%, 100%, 100%, 99.5%, 99.4%, 99.4%, 99.2%, 84.7%, 25.7%, 10.1%, 100%, 100%, 100%	Deafness; autism spectrum intellectual disability; possible anti-atherosclerotic effect	Pathogenic
m.1630A>G (MT-TV)	4	48.3%‡, 7.8%, 3.6%, 2.4%	MNGIE-like disease; MELAS	VUS
m.1644G>A (MT-TV)	4	6.4%, 5.0%, 3.5%, 3.3%	Leigh syndrome; hypertrophic cardiomyopathy; MELAS	Likely pathogenic
m.3242G>A (MT-TL1)	1	4.80%	Mitochondrial myopathy; hypertrophic cardiomyopathy; renal tubular dysfunction*	Likely pathogenic
m.3243A>G (MT-TL1)	1	21.50%	MELAS; Leigh syndrome; diabetes and deafness; MIDD; sensorineural hearing loss; CPEO; mitochondrial myopathy; focal segmental glomerulosclerosis*; autism spectrum disorder; cardiac and multi-organ dysfunction	Pathogenic / Likely pathogenic
m.3255G>A (MT-TL1)	1	4.30%	MERRF; Kearns–Sayre overlap	Likely pathogenic
m.5650G>A (MT-TA)	1	11.10%	Myopathy	Likely pathogenic
m.6930G>A (MT-CO1)	1	3.00%	Multisystem mitochondrial disorder	Likely pathogenic
m.7445A>G (MT-TS1/MT-CO1)	1	19.00%	Sensorineural hearing loss	Pathogenic
m.7453G>A (MT-TS1)	1	9.30%	Fatal neonatal lactic acidosis; neonatal lactic acidosis; exercise	Likely pathogenic

			intolerance; mild intellectual disability	
m.8969G>A (MT-ATP6)	1	22.90%	Mitochondrial myopathy, lactic acidosis, and sideroblastic anemia; IgA nephropathy*	Likely pathogenic
m.9176T>C (MT-ATP6)	1	100%	Familial bilateral striatal necrosis; Leigh disease; spinocerebellar ataxia	Pathogenic
m.10197G>A (MT-ND3)	1	3.60%	Leigh disease; dystonia; stroke-like episodes; LDYT	Pathogenic
m.11778G>A (MT-ND4)	2	100%, 100%	Leber hereditary optic neuropathy; progressive dystonia	Pathogenic
m.12294G>A (MT-TL2)	1	6.50%	Chronic progressive external ophthalmoplegia; external ophthalmoplegia	VUS
m.13513G>A (MT-ND5)	1	2.70%	Leigh disease; MELAS; LHON–MELAS overlap; adult-onset tubulointerstitial nephropathy*; negative association with carotid atherosclerosis	Pathogenic
m.14484T>C (MT-ND6)	2	100% ‡, 90.1%, 19.6%	Leber hereditary optic neuropathy	Pathogenic
m.14568C>T (MT-ND6)	1	99.70%	Leber hereditary optic neuropathy	Likely pathogenic
m.14674T>C (MT-TE)	1	8.70%	Reversible COX deficiency myopathy	Likely pathogenic
m.14710G>A (MT-TE)	1	3.50%	Encephalomyopathy; retinopathy	VUS
m.15242G>A (MT-CYB)	1	2.80%	Mitochondrial encephalomyopathy	Likely pathogenic

Supplement Table 1. Mitochondrial DNA variants identified among kidney disease participants in the Mount Sinai Million cohort

Listed are unique mtDNA variants observed among individuals with kidney disease and no prior primary mitochondrial diagnosis, including sample counts, heteroplasmy levels (reported as individual percentages), reported disease associations, and MITOMAP pathogenicity classifications.

* Renal manifestations previously reported in association with the indicated mtDNA variant.

‡ One individual carried both the m.14484T>C and m.1630A>G variants.

Kidney phenotype (PheCode)	Cases (n)	mtDNA carriers (n)	Carrier frequency (%)
Any kidney disease (GU_580 or GU_582)	3,935	45	1.14
Chronic kidney disease (GU_582.2)	3,374	40	1.19
Renal failure (GU_582)	3,861	44	1.14
Diffuse membranous glomerulonephritis (GU_580.52)	12	1	8.33

Supplementary Table 2. Kidney Disease Subtypes and mtDNA Carrier Frequency

Shown are selected kidney disease subtypes defined by PheCodes and the number and proportion of individuals within each category who carried a clinically significant mitochondrial DNA variant in the Mount Sinai Million cohort. Subtypes shown correspond to those included in the odds ratio–based regression analyses.

STROBE Statement—checklist of items that should be included in reports of observational studies

	Item No.	Recommendation	Page No.	Relevant text from manuscript
**Title and abstract**	1	(*a*) Indicate the study's design with a commonly used term in the title or the abstract	2	
		(*b*) Provide in the abstract an informative and balanced summary of what was done and what was found	3	
**Introduction**				
Background/rationale	2	Explain the scientific background and rationale for the investigation being reported	4,5	
Objectives	3	State specific objectives, including any prespecified hypotheses	5	
**Methods**				
Study design	4	Present key elements of study design early in the paper	5	
Setting	5	Describe the setting, locations, and relevant dates, including periods of recruitment, exposure, follow-up, and data collection	5	
Participants	6	(*a*) *Cohort study*—Give the eligibility criteria, and the sources and methods of selection of participants. Describe methods of follow-up<br>*Case-control study*—Give the eligibility criteria, and the sources and methods of case ascertainment and control selection. Give the rationale for the choice of cases and controls<br>*Cross-sectional study*—Give the eligibility criteria, and the sources and methods of selection of participants	6	
		(*b*) *Cohort study*—For matched studies, give matching criteria and number of exposed and unexposed<br>*Case-control study*—For matched studies, give matching criteria and the number of controls per case	6	
Variables	7	Clearly define all outcomes, exposures, predictors, potential confounders, and effect modifiers. Give diagnostic criteria, if applicable	6	
Data sources/ measurement	8*	For each variable of interest, give sources of data and details of methods of assessment (measurement). Describe comparability of assessment methods if there is more than one group	*5*	
Bias	9	Describe any efforts to address potential sources of bias		
Study size	10	Explain how the study size was arrived at	6	

Continued on next page

Quantitative variables	11	Explain how quantitative variables were handled in the analyses. If applicable, describe which groupings were chosen and why	
Statistical methods	12	(*a*) Describe all statistical methods, including those used to control for confounding	7
		(*b*) Describe any methods used to examine subgroups and interactions	7
		(*c*) Explain how missing data were addressed	7
		(*d*) *Cohort study*—If applicable, explain how loss to follow-up was addressed<br>*Case-control study*—If applicable, explain how matching of cases and controls was addressed<br>*Cross-sectional study*—If applicable, describe analytical methods taking account of sampling strategy	
		(*e*) Describe any sensitivity analyses	
**Results**			
Participants	13*	(a) Report numbers of individuals at each stage of study—eg numbers potentially eligible, examined for eligibility, confirmed eligible, included in the study, completing follow-up, and analysed	7
		(b) Give reasons for non-participation at each stage	
		(c) Consider use of a flow diagram	10 (figure 2)
Descriptive data	14*	(a) Give characteristics of study participants (eg demographic, clinical, social) and information on exposures and potential confounders	7,9, 10.
		(b) Indicate number of participants with missing data for each variable of interest	
		(c) *Cohort study*—Summarise follow-up time (eg, average and total amount)	
Outcome data	15*	*Cohort study*—Report numbers of outcome events or summary measures over time	
		Case-control study—Report numbers in each exposure category, or summary measures of exposure	*7,8,9*
		Cross-sectional study—Report numbers of outcome events or summary measures	
Main results	16	(*a*) Give unadjusted estimates and, if applicable, confounder-adjusted estimates and their precision (eg, 95% confidence interval). Make clear which confounders were adjusted for and why they were included	8,9
		(*b*) Report category boundaries when continuous variables were categorized	10
		(*c*) If relevant, consider translating estimates of relative risk into absolute risk for a meaningful time period	

Continued on next page

Other analyses	17	Report other analyses done—eg analyses of subgroups and interactions, and sensitivity analyses	
**Discussion**			
Key results	18	Summarise key results with reference to study objectives.	10, 11
Limitations	19	Discuss limitations of the study, taking into account sources of potential bias or imprecision. Discuss both direction and magnitude of any potential bias	11,12
Interpretation	20	Give a cautious overall interpretation of results considering objectives, limitations, multiplicity of analyses, results from similar studies, and other relevant evidence	12
Generalisability	21	Discuss the generalisability (external validity) of the study results	12
**Other information**			
Funding	22	Give the source of funding and the role of the funders for the present study and, if applicable, for the original study on which the present article is based	12,13

*Give information separately for cases and controls in case-control studies and, if applicable, for exposed and unexposed groups in cohort and cross-sectional studies.

Note: An Explanation and Elaboration article discusses each checklist item and gives methodological background and published examples of transparent reporting. The STROBE checklist is best used in conjunction with this article (freely available on the Web sites of PLoS Medicine at http://www.plosmedicine.org/, Annals of Internal Medicine at http://www.annals.org/, and Epidemiology at http://www.epidem.com/). Information on the STROBE Initiative is available at www.strobe-statement.org.
